# COVID-19 in children: a case series from Nigeria

**DOI:** 10.11604/pamj.supp.2020.35.2.23597

**Published:** 2020-05-28

**Authors:** Olayinka Rasheed Ibrahim, Bello Mohammed Suleiman, Abdallah Sanda, Taofeek Oloyede, Surajudeen Oyeleke Bello, Umar Ibrahim Bello, Shamsudeen Yahaya, Adamu Dawud, Sulaiman Saidu Bashir

**Affiliations:** 1 Department of Pediatrics, Federal Medical Centre, Katsina, Nigeria; 2 Department of Medical Microbiology, Federal Medical Centre, Katsina, Nigeria; 3 Department of Internal Medicine, Federal Medical Centre, Katsina, Nigeria; 4 Department of Paediatrics, Dalhatu Araf Specialist Hospital, Lafia, Nigeria; 5 Department of Public Health, Katsina State Ministry of Health, Katsina, Nigeria; 6 Department of Disease Control & Immunization, National Primary Health Care Development Agency, Abuja, Nigeria; 7 Department of Community Medicine Amadu Bello University, Zaria, Nigeria

**Keywords:** COVID-19, clinical features, laboratory findings, Nigerian children

## Abstract

**Introduction:**

The global spread of COVID-19 remains unabated in the past few months with a rise in the number of available literature on the novel virus. There are very few paediatric studies and are mainly from developed countries with a paucity of information on the clinical manifestation of COVID-19 disease in African children, including Nigeria.

**Methods:**

We described the clinical presentation, laboratory findings, treatment and outcome in a group of five Nigerian children managed at a COVID-19 isolation and treatment centre in Nigeria.

**Results:**

We managed a total of five children with an age range of 3 months to 8 years in the last four weeks (16th April to 15th May 2020). Three of the five children were males. All the children had close contact with family members that tested positive for COVID-19. Out of the five children, one had moderate disease, three had mild symptomatic disease, and one was asymptomatic. Two out of the five children had lymphocytosis. Out of the four children who had chest radiograph, two had features of pneumonia.

**Conclusion:**

COVID-19 is not uncommon in Nigerian children, and all had a confirmed family member with COVID-19. Besides, contrary to leucopaenia with lymphopaenia observed in the adult’s population, we found lymphocytosis in this cohort and about 50.0% had pneumonic changes on chest radiograph.

## Introduction

The coronavirus disease 2019 (COVID-19) is a ravaging global pandemic that affects both the children and adults [1-3]. Since the first identification of COVID-19 in Wuhan, China, the number of publications on severe acute coronavirus two (SARS-CoV-2) and associated disease has increased [4,5]. However, the available literature is predominantly in adults´ populations, with few paediatric data which affirmed the susceptibility of children to the infectious disease [1]. Besides, the paediatric studies indicated different presentations in term of clinical features, severity and outcomes compared with the adults [2]. A study from Iran indicated variations in the clinical manifestation compared with the data from China among children [6,7]. Besides, the very few paediatric studies were from developed countries with a paucity of studies on the clinical manifestation of COVID-19 disease among African children, including Nigeria [1]. Hence, we described the clinical presentation, laboratory findings, treatment and outcome in a group of five Nigeria children managed at a COVID-19 treatment centre in Nigeria.

## Methods

This study was a retrospective review of a series of five cases, with an age range of 3 months to 8 years. They were brought to the designated COVID-19 treatment centre, in Katsina-Nigeria, between 16th April to 15th May 2020, because they had a positive real-time Polymerase chain reaction (RT-PCR) test for COVID-19. We carried out the COVID-19 confirmatory tests on oropharyngeal and nasopharyngeal swabs at the National Reference Laboratory in Abuja through the real-time polymerase chain reaction. The hospital ethic review committee approved this study, and we obtained informed consent from the parents of the children. We examined demographic variables, clinical presentations, laboratory findings, treatment and outcome in a group of five Nigeria children managed at the centre. The laboratory tests included full blood count and its differentials and chest radiograph. We carried out descriptive analysis using Microsoft Excel version 2016.

## Results

**Figure 1:**
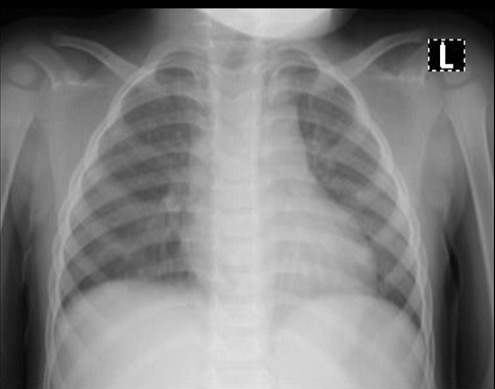
Patient one chest radiograph showing bilateral patchy opacities seen in both lung fields

**Figure 2:**
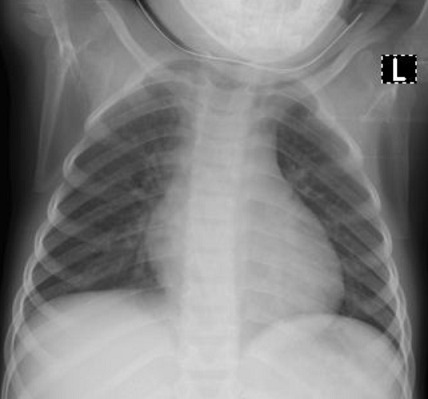
Patient two chest radiograph showing reticular opacities in the paracardiac and paratracheal regions with sparing of the lateral lung fields

**Figure 3:**
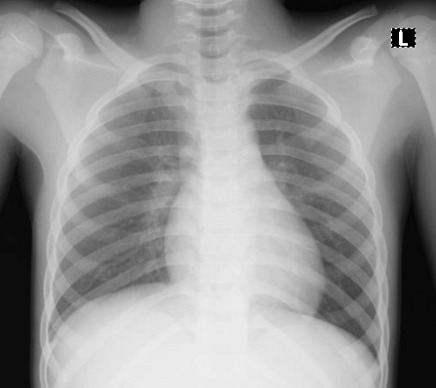
Patient 3 chest radiography showing normal study

Cases 1, 2 and 3 were siblings from the same parents and aged two years, four years and eight years, respectively. Their father had earlier tested positive for SARS-CoV-2. The two-year-old male child presented with a mild cough and nasal discharge. The examinations findings were unremarkable. The total full blood count was within a normal limit (Table 1). However, the chest radiograph showed bilateral patchy opacities in both lung fields (Figure 1). The RT-PCR, which was positive at admission, turned negative after 11 days on admission. The second patient is a four-year-old female who also had a mild cough and nasal discharge. The total full blood count was within a normal limit (Table 1). The chest X-ray showed reticular opacities in the paracardiac and paratracheal regions with sparing of the lateral lung fields (Figure 2). The RT-PCR was positive at admission and turned negative after 13 days on admission. The third patient is an eight-year-old male who presented with a sore throat. The total full blood count was within a normal limit (Table 1), and the chest radiographic findings were normal (Figure 3). The RT-PCR was positive at admission and turned negative after 13 days on admission. All the three children received azithromycin, zinc and lopinavir/ritonavir. Besides, the eight-year-old boy received amoxicillin-clavulanic acid.

**Table 1:** Demographic, clinical presentation, laboratory findings and treatment options of children with COVID-19

Variable	Patient 1	Patient 2	Patient 3	Patient 4	Patient 5
**Age**	2 years	4 years	8 years	7 years	3 months
**Gender**	Male	Female	Male	Female	Male
**Presenting complaints**	Cough Nasal discharge	Cough Nasal discharge	Sore throat	Asymptomatic	Fever Irritability Reduced intake
**Examination findings**	Normal findings	Normal findings	Inflamed tonsils	Normal findings	Febrile (38.40C), splenomegaly
**FBC (X109/l)**	7.7	6.9	8.6	6.8	18.8
**Lymphocyte (%)**	69.3	51.5	50.9	46.6	78.4
**Neutrophil (%)**	17.8	35.6	33.7	38.8	6.8
**Monocytes (%)**	11.1	11.0	12.5	10.8	10.5
**Eosinophils (%)**	1.1	1.8	1.6	3.5	8.9
**Basophils (%)**	0.4	0.1	1.4	0.3	0.4
**PCV (%)**	29.4	28.3	37	36.7	27
**Platelets (X109/l)**	140	210	120	267	217
**Chest radiograph**	bilateral patchy opacities in both lung fields	reticular opacities in the paracardiac and paratracheal regions	normal	N/A	normal
**Other tests**					Na+ -133, K+ -5.6, HCO3-21, Urea 2.4, Cr 41
**Treatment received**	Zinc, lopinavir/ritonavir, azithromycin	Zinc, lopinavir/ritonavir, amoxicillin-clavulanic acid, azithromycin	Zinc, lopinavir/ritonavir, azithromycin	Zinc, lopinavir/ritonavir, azithromycin	Zinc, lopinavir/ritonavir, azithromycin
**Family members positive for Covid-19**	Father	Father	Father	Two older siblings aged 19 and 21, mother	Father

PCV-packed cell volume; N/A (not available); Na+, K+, HCO3, and Urea are in mmol/l; Cr-serum creatinine in μmol/l The SARS-CoV-2 confirmatory tests were done at the National Reference Laboratory in Abuja through the real-time polymerase chain reaction using oropharyngeal and nasopharyngeal swabs.

**Figure 4:**
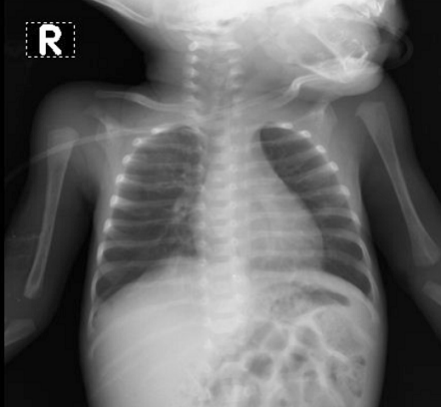
Patient 5 chest radiograph showing normal study

Case four was an asymptomatic seven-year-old girl whose older siblings (aged 19 and 21) and mother tested positive for SARS-CoV-2. The full blood count was normal (Table 1). She received lopinavir/ritonavir and zinc supplementation. The RT-PCR, which was positive at admission, turned negative after nine days. Case five is a three-month-old male infant who presented with two days history of irritability, fever and reduced intake with an initial diagnosis of sepsis. He did not improve on antibiotics which necessitated further evaluation. Further history revealed that the father is a health care worker in a hospital that was managing COVID-19 patients, which prompted screening for COVID-19 in both the child and father. The results of tests for SARS-CoV-2 returned positive for both the child and the father. The full blood count showed leucocytosis with lymphocytosis (Table 1). The chest radiograph done ten days into the illness revealed normal (Figure 4). We revised the diagnosis to moderate COVID-19, and he received tabs zinc, azithromycin and lopinavir/ritonavir with remarkable improvement in the clinical states.

## Discussion

The age range of this study showed that children are vulnerable to COVID-19 across all age categories. This age range also falls within reported ages of children who had COVID-19 in china [8], Iran [6] and other parts of the world [1]. We also observed that all the children in this cohort had family members that tested positive for COVID-19. This finding buttresses the fact that children tend to acquire the disease possibly from the adult´s members of the family who may be asymptomatic. With undocumented transmission from a child to adult, the children in this cohort may have acquired the COVID-19 disease from the adult´s family members [9]. The clinical import is the fact that children with suspected COVID-19 should also include the screening for adults in the family who are potential spreader of the infection to the children. Out of the five children, one had moderate disease, three had mild symptomatic disease, and one was asymptomatic. This observation falls within the global findings that children tend to have asymptomatic or mild disease with good outcome [1]. However, worthy of note is the absence of gastrointestinal symptoms compared with the observation from china. Instead, our study showed the predominance of features of upper respiratory tract infection (URTI), which is similar to the case reports from Iran [6] and Vietnam [10]. Also, the only child with moderate disease had fever suggesting which may be uncommon in children from Africa. There is a need for a high index of suspicions in children with URTI, especially in the area of high transmission or history of a confirmed case of COVID-19 in the family.

The main laboratory finding in our study is lymphocytosis (two out of five), which contrast with the observation of leucopaenia with lymphopaenia reported among the adult’s population [11]. The ingress of this finding is the need to carefully interpret the haematological profile of children in areas of high transmission, and the presence of lymphocytosis may prompt screening for COVID-19 disease. Out of the four children who had chest radiograph, two had features of pneumonia which further support the earlier study that mild disease in children with COVID-19 has some radiological findings [12]. The child with the moderate disease had a normal x-ray which may be due to the timing of the radiography (done ten days into the illness) with a possible resolution of early pneumonic changes. With no definitive treatment for COVID-19, the children received zinc, lopinavir/ritonavir, and azithromycin with improvement in their clinical state. The choice of these medications was based on compassionate ground and well-established safety profile in children. Except for the infant who did not tolerate the lopinavir/ritonavir pellets, other children took the drugs without any observed untold side effects. Other studies also administered similar drugs, most of which were given on compassionate ground as the world await the outcome of clinical trials and vaccine development [6,10].

## Conclusion

This study showed that the COVID-19 is not uncommon in Nigerian children and infected family members are potential sources of infection of children with COVID-19. Besides, contrary to leucopaenia with lymphopaenia observed in the adult’s population, we found lymphocytosis in this cohort, and 50.0% had pneumonic changes on chest radiograph..

## Competing interests

The author declares no competing interests.
